# Understanding Language Reorganization With Neuroimaging: How Language Adapts to Different Focal Lesions and Insights Into Clinical Applications

**DOI:** 10.3389/fnhum.2022.747215

**Published:** 2022-02-18

**Authors:** Luca Pasquini, Alberto Di Napoli, Maria Camilla Rossi-Espagnet, Emiliano Visconti, Antonio Napolitano, Andrea Romano, Alessandro Bozzao, Kyung K. Peck, Andrei I. Holodny

**Affiliations:** ^1^Neuroradiology Service, Department of Radiology, Memorial Sloan Kettering Cancer Center, New York, NY, United States; ^2^Neuroradiology Unit, NESMOS Department, Sant’Andrea Hospital, La Sapienza University, Rome, Italy; ^3^Radiology Department, Castelli Hospital, Rome, Italy; ^4^IRCCS Fondazione Santa Lucia, Rome, Italy; ^5^Neuroradiology Unit, Imaging Department, Bambino Gesù Children’s Hospital, Rome, Italy; ^6^Neuroradiology Unit, Cesena Surgery and Trauma Department, M. Bufalini Hospital, AUSL Romagna, Cesena, Italy; ^7^Medical Physics Department, Bambino Gesù Children’s Hospital, Rome, Italy; ^8^Department of Medical Physics, Memorial Sloan Kettering Cancer Center, New York, NY, United States; ^9^Department of Radiology, Weill Medical College of Cornell University, New York, NY, United States; ^10^Department of Neuroscience, Weill-Cornell Graduate School of the Medical Sciences, New York, NY, United States

**Keywords:** language, tumor, epilepsy, stroke, fMRI, DTI—diffusion tensor imaging, reorganization, plasticity

## Abstract

When the language-dominant hemisphere is damaged by a focal lesion, the brain may reorganize the language network through functional and structural changes known as adaptive plasticity. Adaptive plasticity is documented for triggers including ischemic, tumoral, and epileptic focal lesions, with effects in clinical practice. Many questions remain regarding language plasticity. Different lesions may induce different patterns of reorganization depending on pathologic features, location in the brain, and timing of onset. Neuroimaging provides insights into language plasticity due to its non-invasiveness, ability to image the whole brain, and large-scale implementation. This review provides an overview of language plasticity on MRI with insights for patient care. First, we describe the structural and functional language network as depicted by neuroimaging. Second, we explore language reorganization triggered by stroke, brain tumors, and epileptic lesions and analyze applications in clinical diagnosis and treatment planning. By comparing different focal lesions, we investigate determinants of language plasticity including lesion location and timing of onset, longitudinal evolution of reorganization, and the relationship between structural and functional changes.

## Introduction

Functional activations in the superior temporal cortex and inferior supramarginal gyrus (SMG) are traditionally considered to serve speech comprehension [Wernicke’s area (WA)], while the inferior frontal gyrus is thought to serve speech production [Broca’s area (BA)] (Friederici and Gierhan, 2013). In reality, the system is far more complicated. Language processing involves a modular architecture that includes both cortical and subcortical components, distributed anatomical connectivity, and domain-general networks (Tremblay and Dick, 2016). In the “hodotopic” model proposed by Herbet and Duffau (2020), cognitive functions like language rely on dynamic interactions between cortical hubs and subcortical white matter connections. These interactions can be captured by neuroimaging, particularly through advanced MRI techniques that can show cortical activations [functional magnetic resonance imaging fMRI)] and subcortical structural connections [diffusion tensor imaging DTI)] (Pan et al., 2012; Jenabi et al., 2014, 2015).

According to Herbet and Duffau (2020), the brain is not a mosaic of independent modules, but is organized into parallel networks that compensate for one another. The ability to compensate and reorganize underlies the phenomenon known as brain plasticity. The first observations of this phenomenon can be traced back to the beginning of the 20th century with Ramón y Cajal, followed by the studies of Hebb (1949) more than 70 years ago, which reported that coincident neuronal activity may lead to structural changes and synapse strengthening (Stahnisch and Nitsch, 2002; Tillet and Chalon, 2018). Regarding language function, when the dominant hemisphere is damaged by a focal lesion, the brain may reorganize by means of functional and structural changes. This phenomenon has been documented for different triggers, including stroke, fast and slow-growing tumors, and epilepsy (Holodny et al., 2002; Petrovich et al., 2004; Mbwana et al., 2009; Rosenberger et al., 2009; Turkeltaub et al., 2011; Labudda et al., 2012; Krieg et al., 2013; Li et al., 2019).

Many questions remain unanswered regarding the effect of focal lesions on language plasticity. The time pattern of a lesion is thought to play a pivotal role in the dynamic of reorganization (Desmurget et al., 2007). However, comparative studies of slow onset (such as low-grade tumors) vs. rapid onset lesions (such as stroke or fast growing tumors) are scarce in the literature. Different focal lesions may induce different patterns of reorganization depending on specific pathologic mechanisms or location in the brain. Finally, considerations regarding language reorganization are often separated from the diagnosis and treatment of actual patients and are therefore seldom assessed on clinical imaging. For example, in the presurgical planning of brain tumors, fMRI is primarily used for language dominance (Brennan et al., 2016) although the same technique can also provide insights into brain connectivity (Bullmore and Sporns, 2009; Li et al., 2020, 2021). Similarly, stroke patients rarely undergo fMRI in clinical practice, foregoing useful information to understand functional deficits and to provide individualized rehabilitation (Kiran and Thompson, 2019). This leads to the important issue of how best to evaluate language plasticity and obtain plasticity information without disrupting the standard diagnostic workflow. fMRI appears to be a good candidate to solve this problem due to its non-invasive nature, ability to image the whole brain, and large-scale implementation in clinical practice. These advantages make fMRI a valuable technique to provide insights into brain plasticity (Gupta et al., 2010).

This paper provides an overview of language reorganization on neuroimaging, particularly MRI. Our objective is to describe the ways in which language plasticity is visualized on brain scans obtained in the clinical practice, to provide a reference for radiologists and clinicians dealing with brain imaging. First, we describe the language network as depicted by DTI (structural network) and fMRI (functional network). Second, we explore language reorganization triggered by focal brain lesions and current clinical applications. Our comparison of stroke and brain tumors highlights neuroimaging’s potential to disentangle the many factors influencing language plasticity, including the effects of lesion location, onset, and timing. It also sheds light on the multiple mechanisms of brain plasticity itself, such as longitudinal evolution of reorganization and the relationship between structural and functional changes.

## The Language Network

The language network can be viewed from a structural or a functional perspective. Understanding the organization of language function in a healthy individual allows for appreciation of language reorganization in pathological conditions.

### The Structural Language Network

In the vast majority of the adult population, the eloquent language cortex is distributed in the left frontal and superior temporal lobes, including the opercular and triangular part of the inferior frontal gyrus (op-IFG and tr-IFG respectively), traditionally known as Broca’s area (BA) (Friederici and Gierhan, 2013) and the posterior aspect of the superior temporal gyrus (STG), traditionally known as Wernicke’s area (WA) (Friederici and Gierhan, 2013). The premotor cortex (Brodmann’s area 6) covers a vast area of the frontal lobe anterior to the motor cortex (Brodmann’s area 4), including the anterior and lateral precentral gyrus, posterior aspect of the middle frontal gyrus (MFG), and posterior aspect of the superior frontal gyrus (SFG) (Duffau et al., 2003a). This important region, herein referred to as premotor area (PreMA), encompasses other eloquent language areas, as well. The other eloquent language areas include the pre-supplementary motor area (pre-SMA), also known as language supplementary motor area (SMA), located in the dorsal and the medial aspect of the SFG (Hertrich et al., 2016); Exner’s area (EA), located in the posterior aspect of the MFG (Roux et al., 2010); and ventral PreMA, corresponding to the anterior/inferior cortex of the precentral gyrus and typically associated with articulatory planning and demonstrating negative motor responses when electrically stimulated (Petrovich et al., 2005; Rech et al., 2019).

**FIGURE 1:**
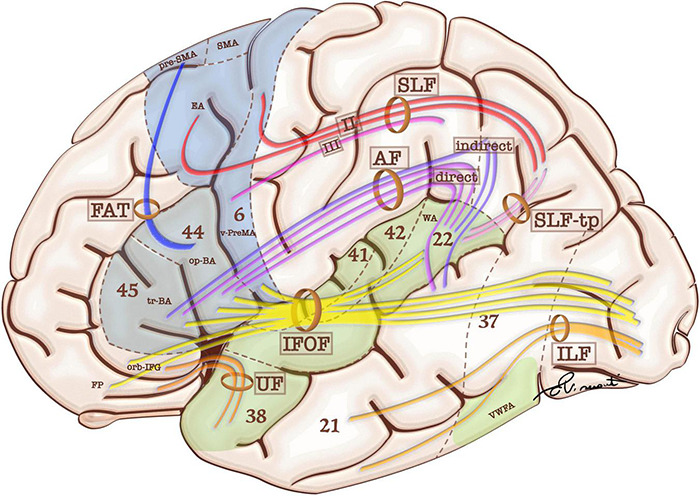
Schematic representation of language-relevant cortical areas and white matter tracts comprising the functional and structural language network. Blue areas are more involved in language production, while green areas are more involved in language comprehension. White matter tracts include frontal aslant tract (FAT), superior longitudinal fasciculus (SFL and SFL-tp for the temporal part), and arcuate fasciculus (AF). These bundles participate in the dorsal stream of language. The ventral stream of language includes: uncinate fasciculus (UF), inferior longitudinal fasciculus (ILF), and inferior frontal-occipital fasciculus (IFOF). Language-relevant cortical areas include Broca’s area in the triangular (tr-BA) and opercular (op-BA) part of the inferior frontal gyrus (Brodmann’s areas 45 and 44 respectively); the pre-supplementary motor area (pre-SMA), Exner’s area (EA) and ventral premotor area (v-PreMA) in the premotor cortex (Brodmann’s area 6); Wernicke’s area (WA) in the superior temporal gyrus (Brodmann’s area 22).

Eloquent cortical regions are connected by white matter bundles. According to previous studies, human language relies on two primary white matter pathways: the dorsal stream and the ventral stream. These are related to sensorimotor integration and speech comprehension respectively (Figure 1; Dick et al., 2014; Chang et al., 2015). The dorsal stream is formed by white matter fibers of the superior longitudinal fasciculus (SLF)/arcuate fasciculus (AF) system, which is considered a single white matter bundle with multiple subcomponents: AF, SLF I, II, III and SLF-tp. The AF connects the op-IFG and tr-IFG of the inferior frontal gyrus and the ventral premotor area (PreMA) to the posterior STG and middle temporal gyrus (MTG). The AF is composed of two pathways (Figure 1). The AF direct pathway is located medially and represents a direct connection between BA and WA. The AF indirect pathway runs laterally and is formed by both an anterior segment linking the inferior parietal cortex to BA, and a posterior segment connecting the inferior parietal cortex to WA (Catani et al., 2005). The SLF I, II, and III join the frontal and parietal cortices, while the SLF-tp subcomponent joins the temporal and parietal cortices. SLF II, III, and tp represent the eloquent components for language and are discussed in further detail below.

SLF II connects the dorsal premotor cortex to the angular gyrus (AG), while the SLF III connects the ventral PreMA and op-IFG to the SMG. The SLF-tp is a small tract which connects the AG to the posterior aspect of the STG (Dick et al., 2014; Chang et al., 2015). The frontal aslant tract (FAT) connects the SMA and pre-SMA to the op-IFG and tr-IFG, serving the verbal fluency components of language (Catani et al., 2013; Jenabi et al., 2014). Sensorimotor integration culminates in the op-BA area and ventral PreMA, which are responsible for articulatory planning (Petrovich et al., 2005; Rech et al., 2019). The ventral PreMA represents a crucial speech production hub thanks to its coupling with the SLF III. Preservation of this cortical-subcortical connection is crucial for speech integrity and represents an anatomical constraint to cortical plasticity (van Geemen et al., 2014).

The most widely accepted description for the ventral pathway includes two major fasciculi: the inferior frontal-occipital fasciculus (IFOF) and the uncinated fasciculus (UF) (Dick et al., 2014; Chang et al., 2015). The IFOF originates in the inferior/medial occipital lobe and posterior STG. It sends projections to the ventral temporal lobe and terminates in the IFG, orbital frontal cortex, and frontal pole. The UF connects the anterior temporal lobe and the inferior frontal areas (Dick et al., 2014; Chang et al., 2015). According to Duffau et al. (2013), the IFOF constitutes the primary pathway for the ventral stream (direct pathway), accounting for the semantic processing of language, while the UF and the inferior longitudinal fasciculus (ILF) represent a secondary (indirect) pathway that is believed to play a role in representing emotional valence (Mandonnet, 2017). While the direct pathway of the ventral stream is deemed pivotal for language, the indirect pathway is considered more expendable for surgical purposes (Duffau et al., 2013). Direct cortical stimulation (DCS) studies have confirmed that perceived speech inputs are transferred via direct AF fibers from the posterior two thirds of STG/MTG (mainly representing WA) to the IFG (particularly op-IFG and tr-IFG) to be converted into working phonological–phonetic representations. This information is then translated into articulatory motor outputs mediated by the ventral PreMA (Sarubbo et al., 2020).

### The Functional Language Network From tb-fMRI

**FIGURE 2:**
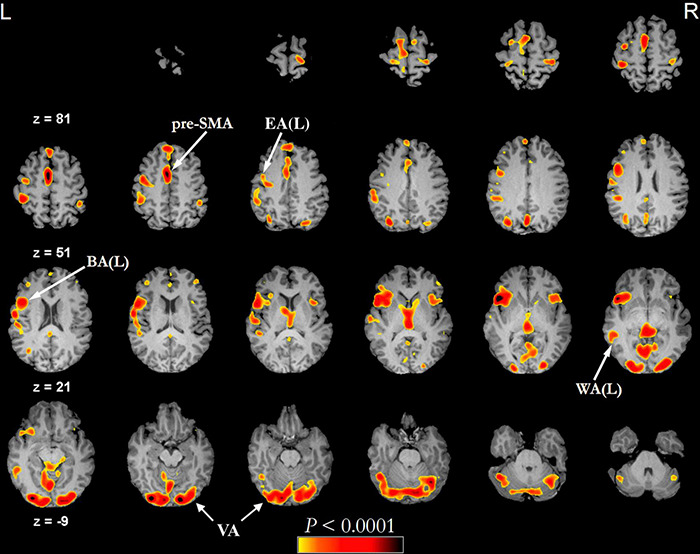
Example of functional activations seen on a phonemic fluency language task (threshold of minimum correlation *r* = 0.5 (uncorrected *p* < 0.0001), minimum cluster size of 20 voxels (1280 mm^3^) administered visually in a healthy subject (43 years old male). In this type of task, the subject is asked to silently generate words starting with a specific letter. The fMRI language activation map is overlayed on the subject’s pre-contrast T1-weighted 3d images after skull stripping. BA, Broca’s area; EA, Exner’s area; pre-SMA, pre-supplementary motor area; VA, visual activation; WA, Wernicke’s area.

Recently, researchers have reconsidered the functional localization of speech hubs (Tate et al., 2014) and noted relevant variability in the classical description of BA and WA (Sanai et al., 2008; Tremblay and Dick, 2016). However, current clinical practice still benefits from localization of eloquent areas both prior to surgical procedures and to monitor recovery. The most widely employed non-invasive method to evaluate language function in clinical practice is task-based fMRI (tb-fMRI) (Smits et al., 2006; Brennan et al., 2016). The functional network obtained from this technique differs slightly depending on the employed language task. In visually-administered tasks (Figure 2), the information travels from the visual cortex (occipital lobe around the calcarine fissure), frontal eye fields, and Exner’s area (Matsuo et al., 2003; Roux et al., 2010) to a “common language network” (Benjamin et al., 2017), which is relevant in clinical practice and contains most of the areas localized for treatment planning: BA, WA, and pre-SMA (Lyo et al., 2015). Exner’s area is located in the posterior aspect of the middle frontal gyrus as part of the PreMA (Brodmann’s area 6). This area is critically involved in transforming phonological representations of words into motor commands for handwriting (Roux et al., 2010). It also participates in both naming (Corrivetti et al., 2019) and reading (Pattamadilok et al., 2016). The frontal eye field is a bilateral activation located in Brodmann’s area 6 in humans, very close to Exner’s area, and that serves saccadic eye movements for the purpose of visual field perception and awareness, as well as voluntary eye movement. To distinguish these two areas, we rely on lateralization (Exner’s area is often lateralized in the dominant hemisphere) (Dong et al., 2016) or specific tasks (Matsuo et al., 2003). If a visually administered task includes reading, activation of the visual word form area (VWFA) located in the inferior temporal gyrus (ITG) and fusiform gyrus is expected. This area is involved in the elaboration of visual information, integrating inputs from the ipsilateral and contralateral occipital poles (Mandonnet, 2017; Sarubbo et al., 2020). Additionally, the VWFA has been related to a range of language tasks, including visual and auditory naming, auditory comprehension, repetition, and spontaneous speech (Benjamin et al., 2017). Aurally administered tasks are characterized by activation of the auditory network centered in the Heschl’s gyrus (primary auditory cortex) (da Costa et al., 2011), which leads to the “common language network” without visual activation. Other differences arise between different task typology. According to the recommendations of the American Society of Functional Neuroradiology (ASFNR), an adult language paradigm algorithm for presurgical language assessment should include the combination of sentence completion and silent word generation tasks (Black et al., 2017). Silent word generation is a verbal fluency task that requires phonologic access, verbal working memory, and lexical search activity. These result in strong activation and lateralization of frontal areas (Zacà et al., 2013; Corrivetti et al., 2019; Emch et al., 2019). Sentence completion requires word recognition and comprehension, understanding of syntactic–semantic relationships between words, planning of a sentence structure, and word retrieval, with diffused activation of the lexical-semantic network (Li et al., 2017). These lead to decreased language lateralization and increased recruitment of secondary language areas, but also to a better identification of WA (Unadkat et al., 2019).

Secondary language areas are variably active in different tasks. The SMG is involved in auditory attention and categorization functions that are not specific to speech (Price, 2012). It also participates in the widespread network that supports bottom-up attention (Corbetta et al., 2008). The AG plays a role in semantic processing of written words and pictures (Price, 2012). The left insula is involved in syntactic processing, articulatory planning, and phonation (Price, 2012). The cerebellum plays an important role in motor speech control, however more recent evidence supports its participation in speech perception and high-order linguistic processes such as speech timing and phonological aspects of lexical access (Mariën et al., 2014). Particularly, the cerebellar lobules VI and Crus I seem to hold motor representations of phonological content, while the inferior cerebellar lobules VIIb/VIII may provide phonological storage during the maintenance of verbal information (Mariën et al., 2014). On clinical fMRI, cerebellar activation is often lateralized to the right (Cho et al., 2018), as opposed to main language areas. The production/understanding of language may receive contributions from the hippocampal declarative memory system (Duff and Brown-Schmidt, 2012). According to this hypothesis, speakers and listeners use the hippocampal memory system to generate, gather, integrate, and maintain representations that serve language processing. The hippocampus has also been implicated in the ability to simulate and predict future events (Covington and Duff, 2016), drawing memory representations of upcoming words while language unfolds over time.

Secondary language areas are often considered to be less important than primary areas and expendable in clinical practice, including surgical planning (Sarubbo et al., 2020). However, fMRI standard analysis is not capable of discerning which language area is more important for the stability of the network (and should be surgically spared) since it cannot describe their hierarchical relationship. The application of graph theory to fMRI can help us to better understand the concept of hierarchical organization of language areas by identifying “core” network components whose integrity is essential to network stability (Morone and Makse, 2015; del Ferraro et al., 2018; Li et al., 2020). Centrality measures can highlight crucial nodes in a network by testing network stability through the progressive removal of links until architectural collapse (Morone et al., 2019). Application of these techniques has shown that pre-SMA, PreMA, and BA are part of a frontal “core” of the human language network (Li et al., 2020) that is tightly connected to WA and remains consistent across healthy patients regardless of their spoken language (Li et al., 2021).

## The Plastic Potential of the Brain

**FIGURE 3:**
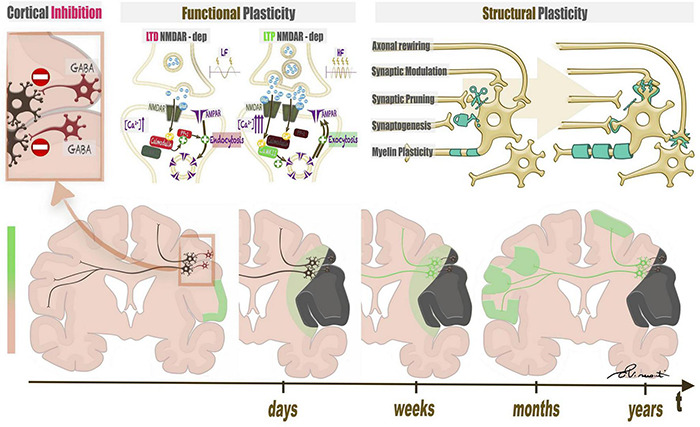
Structural and functional plasticity of language triggered by a focal lesion. The left upper panel represents the baseline condition of the brain, characterized by widespread inhibition mediated by GABA interneurons. When a lesion attacks eloquent brain areas, the damage triggers disinhibition of nearby neural networks, possibly leading to functional and structural plasticity. Functional plasticity **(central upper panel)** at the synaptic level consists of long term potentiation (LTP) or depotentiation (LTD), which depend on an increase in calcium ions in the post-synaptic neurons of inhibitory and excitatory synapses. Structural plasticity **(right upper panel)** includes changes in synapses, axons, and myelin coating. The lower panel represents a timeline of plastic changes in the brain. After lesion onset, perilesional and contralateral modifications follow.

The term “brain plasticity” refers to two distinct processes, functional plasticity and structural plasticity. Functional plasticity involves variation of synaptic strengths without changing the anatomical connectivity between neurons while structural plasticity requires anatomical changes (Figure 3). Functional plasticity at the synaptic level consists of long term potentiation (LTP) or depotentiation (LTD), defined as persistent strengthening/weakening of synapses based on recent patterns of activity (Dan and Poo, 2004; Ganguly and Poo, 2013). Structural plasticity includes separate strategies. The first, synaptogenesis, is defined as change in synaptic wiring schemes through the formation of new synapses. Retraction and reformation of dendritic spines encompasses re-routing of axonal branches (axonal rewiring) (Butz et al., 2009). Synaptic pruning consists of synapse number optimization to prioritize most effective connections (Sakai, 2020). Myelin plasticity encompasses variations of oligodendrocyte proliferation and differentiation, changes in nodal or internodal length, as well as myelin remodeling (Sampaio-Baptista and Johansen-Berg, 2017). Neurogenesis is the last form of structural plasticity, although its role in the adult brain is still debated (Kempermann et al., 2004; Sailor et al., 2017). Neuronal activity, whether spontaneous or driven by sensory experience, is a link between structural and functional plasticity and is important for the formation of synapses. Transmitter release of an intensely activated area may promote synapse formation and cortical rewiring (Andreae and Burrone, 2014). In turn, changes in synaptic number and morphology may accompany LTP/LTD (Ganguly and Poo, 2013).

All of the above-mentioned processes translate to macroscopic changes in brain anatomy and function, which may be detectable on MRI. Blood oxygenation dependent (BOLD) signal increases may result from microscopic changes at the neurovascular unit level. Neurons regulate cerebral blood flow (CBF) by generating signals that induce local blood vessels to initiate the vascular response. Glutamatergic synaptic activity activates a post-synaptic cascade leading to activation of neuronal NO synthase (nNOS) and cyclooxygenase 2 (COX-2), which produce potent vasodilators (Iadecola, 2017). At the same time, glutamatergic activity participates in LTP, justifying the link between microscopic and macroscopic functional changes (Dhuriya and Sharma, 2020). The learning of new functions leads to structural plasticity of involved brain areas and changes in cortical volume or thickness, possibly reflecting synaptic rewiring (Draganski et al., 2004, 2006). Anatomical changes in the cortex are detected by MRI through conventional images such as volumetric T1-weighted sequences with the appropriate post-processing (Takao et al., 2010). Myelin remodeling and axonal modifications affect the diffusion parameters of brain tissue and can be studied using MRI techniques, such as DTI or neurite orientation dispersion and density imaging (NODDI) (Timmers et al., 2016; Sato et al., 2017).

Neuroplasticity in focal lesions relies on the interplay between energy levels in the cortex. During normal development, cortical inhibition via gamma-aminobutyric acid (GABA) interneurons reaches a time window known as the “critical period” (CP) when physiologic plasticity may occur. In the adult brain, inhibition grows until the CP window closes to protect established neural circuits from undesired changes and to maintain stored memories and learned skills (Nahmani and Turrigiano, 2014; Ribic et al., 2019). Cortical inhibition is widespread in the adult brain. Most firing activity in brains of anesthetized and awake animals occurs in less than 10% of all neurons (Ovsepian, 2019). After injury, the insult causes cell death, synaptic loss, and decreased GABAergic output, which lead to increased excitability, neurite expansion, and synapse gain (Dancause et al., 2005). Cortical disinhibition unmasks subthreshold inputs, creating a permissive environment for the activation of nearby circuits (Nahmani and Turrigiano, 2014). When a lesion attacks eloquent brain areas, the damage triggers disinhibition of nearby neural networks, possibly leading to adaptive plasticity. In the case of language function, healthy right-handed subjects normally show left-hemispheric dominance (91–96%) with a small minority of subjects showing native co-dominance or right dominance (atypical dominance for language) (Knecht et al., 2000; Isaacs et al., 2006). When the left dominant hemisphere is compromised by a focal lesion such as stroke, tumor, or an epileptic focus, the brain may reorganize the language function in different fashions. Intra-hemispheric reorganization consists of the recruitment of nearby circuits shared with other functions (Hartwigsen et al., 2017). Contralateral reorganization relies on the right hemisphere, likely recruited by inter-hemispheric disinhibition *via* the corpus callosum (Butz et al., 2009; Tantillo et al., 2016).

## Language Plasticity Triggered by Brain Tumors

Despite remarkable efforts to better understand language plasticity, this phenomenon remains mostly isolated from clinical practice. Particularly, the interdependence of active fMRI clusters in the context of functional reorganization remains a major interrogative in neuroscience, with open clinical applications.

### Structural Plasticity in Brain Tumors

Structural plasticity in the setting of brain tumors may manifest at the cortical or subcortical level. LGG growing in the left insula showed increased cortical volume of the contralateral cortex (Almairac et al., 2018) as demonstrated by voxel-based morphometry (VBM) on MRI. Similarly, Zhang et al. (2018) demonstrated increased cortical volume in cerebellar regions of LGG patients. Possible etiologies may include increased cell size or spine density, neural or glial cell genesis, or a combination of slow evolving processes such as myelin plasticity, axonal sprouting, or angiogenesis (Almairac et al., 2018). Subcortical paths are less prone to plastic changes than are cortical areas. While cortical areas often allow resection, white matter bundles constrain surgical radicality (Ius et al., 2011; Picart et al., 2019; Sarubbo et al., 2020). Nevertheless, structural modification of white matter paths has been associated with brain tumors. de Witte et al. (2014) reported higher fiber density, length, and fractional anisotropy (FA) in the right AF of a right-handed patient harboring a left-hemispheric LGG. The patient displayed atypical language dominance on fMRI and lack of speech arrest at DCS (de Witte et al., 2014). In a larger study, patients with left-hemispheric gliomas and no language deficits displayed right-lateralized AF, whereas patients with language deficits demonstrated left-lateralization (Jehna et al., 2017). Interestingly, the laterality index of the posterior segment of the AF was one of the best predictors of language deficits in this study, supporting the importance of this white matter bundle in language processing and reorganization (Jehna et al., 2017). In a study by Zheng et al. (2013), ventral language pathways (left ILF and left IFOF) were considered to take on compensatory functions in patients with left frontal tumors invading the dominant AF, as depicted by FA increases compared with healthy subjects. Within the ventral stream, the IFOF seems to play a pivotal role in compensating damage to the indirect pathways (ILF, UF) as demonstrated by DCS during awake surgery (Duffau et al., 2013). Tantillo et al. reported increased inter-hemispheric transfer of information in right-handed patients harboring left-hemispheric gliomas; atypical language lateralization was correlated with more directional microstructure of the corpus callosum as depicted by increased FA (Tantillo et al., 2016). This may suggest that inter-hemispheric transfer via the corpus callosum underlies language reorganization on the right side (Butz et al., 2009; Tantillo et al., 2016). White matter can be evaluated in a whole-brain fashion by connectomics (Hagmann et al., 2007; Sporns, 2013). Patients harboring brain tumors showed different connectomes than healthy controls. For example, Yu et al. (2016) reported increased small-worldness in patients with brain tumors, possibly related to anatomical changes.

Structural plasticity induced by brain tumors seems to follow intuitive time constraints, with slow-growing neoplasm allowing for wider changes (Desmurget et al., 2007). Nevertheless, structural modifications have been reported in HGG (Jehna et al., 2017). This may seem counterintuitive at first and may be explained by learning-related structural plasticity in healthy patients. For example, modifications of cortical volume after learning a new language may develop within three months (Mårtensson et al., 2012), while the median age of glioblastomas at diagnosis is approximately 330 days (Stensjøen et al., 2018). Despite this evidence, the topic remains an avenue for future research.

Structural plasticity has clinical correlates. After resection of left perisylvian gliomas, patient deficits correlated with the number of fibers detected in the IFOF, FAT, SLF, and AF on longitudinal follow-up (Ille et al., 2018). Furthermore, brain tumors contacting the periventricular zone (PVZ) demonstrated worse prognosis than others (Mistry et al., 2017). This may depend on factors including higher chance of ependymal dissemination or richer peritumoral environment within the stem-cell niche (Sinnaeve et al., 2018). Although there is not enough evidence to support a definite conclusion, neurogenesis impairment may determine worse prognosis by limiting plastic phenomena.

Future studies should investigate white matter modifications by means of more sophisticated techniques such as radial/axial diffusivity from DTI and neurite dispersion from NODDI, which showed impressive concordance with pathologic studies in depicting myelin alteration (Timmers et al., 2016; Sato et al., 2017).

### Functional Plasticity in Brain Tumors

Studies using resting state fMRI (rs-fMRI) have reported that, despite different onset timing, both low- and high-grade left-hemispheric gliomas can affect the functional connectivity of language-related areas with long-distance effects on the contralateral hemisphere (Briganti et al., 2012). Similar results have been obtained with magnetoencephalography (MEG). Traut et al. demonstrated a change in language laterality from left or right to co-dominant in 23.3% of patients with LGGs and HGGs. A complete contralateral shift was present in 5.5% of subjects. Notably, patients with tumors affecting the dominant hemisphere experienced significantly greater changes in language laterality than those with tumors in the non-dominant hemisphere (Traut et al., 2019). The measure to which such alterations represent compensatory reorganization (adaptive plasticity) is still debated. Nevertheless, electrophysiologic studies provided some evidence in support of a partial compensatory nature. Patients with brain tumors in the language-dominant hemisphere showed a neuronal response in the right hemisphere similar to that of left-sided language eloquent areas on MEG, including timing and amplitude modulation. This evidence seems to support the hypothesis that right-hemispheric reorganization is similar in nature to normal processes performed by the left hemisphere (Piai et al., 2020).

**FIGURE 4:**
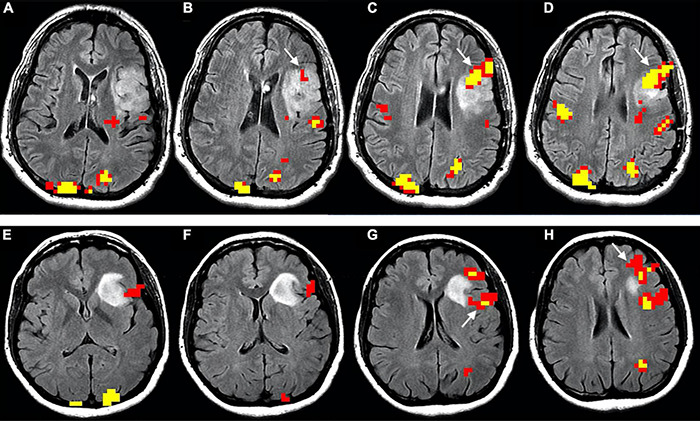
Functional reorganization of language in two right-handed patients with low-grade glioma invading the left inferior frontal gyrus, displayed as functional overlay of phonemic fluency task on axial FLAIR images. The images above **(A–D)** show functional activation inside the tumor (white arrows) in a 50-year-old male, corresponding to the expected location of BA. The images below **(E–H)** display functional activation in a 38-year-old female. The activation surrounds the tumor in the opercular Broca’s area [white arrow in panel **(G)**] and anterior portion of the inferior frontal gyrus, extending to the middle frontal gyrus [white arrow in panel **(H)**]. Functional activation maps associated with the phonemic fluency task were generated at a threshold of minimum correlation *r* = 0.5 (uncorrected *p* = 2 × 10^– 11^). Voxels showing a minimum cluster size of 20 voxels (1,280 mm^3^) were selected.

**FIGURE 5:**
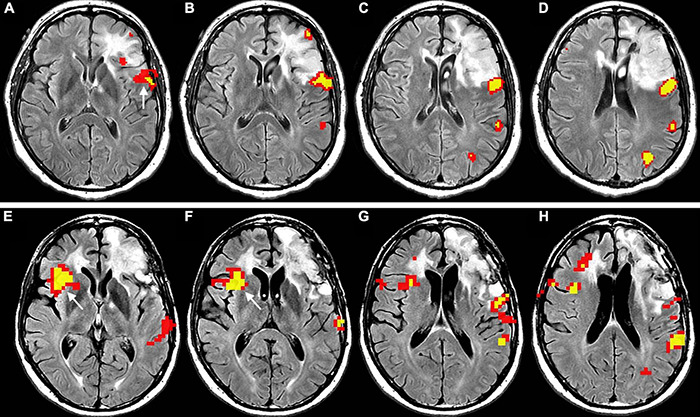
Longitudinal representation of language plasticity in a 43-year-old right-handed male patient with low-grade glioma invading the left inferior frontal gyrus, displayed as functional overlay of phonemic fluency task on axial FLAIR images. The images above **(A–D)**, obtained in the preoperative setting, show functional activation surrounding the tumor in the inferior frontal gyrus, including the expected location of Broca’s area (white arrow in panel **(A)**]. The images below **(E–H)** were obtained at four-year follow-up before a second surgery. A new strong functional activation is visible in the right inferior frontal gyrus, corresponding to Broca’s area homologue (white arrow in panels **(E,F)**]. The patient underwent awake surgery and direct cortical stimulation of the left inferior frontal gyrus, which demonstrated absence of speech arrest. This finding confirms compensatory shift of language representation to the right side. Functional activation maps associated with the phonemic fluency task were generated at a threshold of minimum correlation *r* = 0.5 (uncorrected *p* = 2 × 10^– 11^). Voxels showing a minimum cluster size of 20 voxels (1,280 mm^3^) were selected.

Language functional reorganization in the setting of brain tumors includes perilesional rearrangement (Figure 4; Duffau et al., 2003b; Desmurget et al., 2007), as well as recruitment of contralateral language area homologues (Figure 5; Holodny et al., 2002; Petrovich et al., 2004; Rosenberg et al., 2008; Wang et al., 2013; Gȩbska-Kośla et al., 2017; Li et al., 2019). The process seems to be influenced by tumor pathology (Duffau, 2017) and genetics (Salek et al., 2017), as less aggressive behavior may facilitate plastic phenomena. The slower pace of growth of low-grade versus high-grade tumors is considered an important factor in the allowance of plastic changes in the brain (Desmurget et al., 2007). Similarly, tumor genetics reflects growth dynamics and may influence brain plasticity. Mutations such as isocitrate dehydrogenase (IDH) in gliomas relate to lower aggressivity and longer survival (Louis et al., 2020). Additionally, the location of a lesion does influence the outcome. Damage to brain regions important for communication between subnetworks (connectors) causes greater effects than does damage to peripheral areas (Gratton et al., 2012; Shaw et al., 2016). One of the primary theories to frame the complex scenario of tumor-induced plasticity considers the pace of growth as the main determinant of functional reorganization and was developed by studying LGG (Duffau, 2005).

Functional plasticity may follow a progressive pattern, shifting from within the tumor to the immediate surroundings, and later to more distant areas (Desmurget et al., 2007). For example, non-aphasic patients with BA gliomas may exhibit language-related activation in the left peritumoral inferior frontal cortex (Meyer et al., 2003b). Tumors in the left insula present perisylvian compensatory activation (Duffau et al., 2001). Similarly, patients with left-hemispheric tumors showed increased activation in left Brodmann’s area 46, Brodmann’s area 47, anterior insula, and left cerebellum when compared to healthy controls (Thiel et al., 2001). More specifically, patients with LGGs invading BA demonstrated plastic activations in at least one area among the left insula, premotor cortex, and pars orbitalis of the IFG (Brodmann’s 47) (Benzagmount et al., 2007). After that, a distributed network of areas can be recruited within the contralateral hemisphere. This includes translocation of BA (Holodny et al., 2002; Gȩbska-Kośla et al., 2017; Li et al., 2019) or WA to the right hemisphere (Petrovich et al., 2004; Kośla et al., 2012). Rosenberg et al. (2008) described a lateralization shift to the right side (atypical dominance) during the two years preceding surgery in a right-handed patient with left-hemispheric LGG.

Connectivity metrics may help us to better understand the network modifications underlying language reorganization. Li et al. (2019) described a patient with functional translocation of BA to the right side. As opposed to the functional connection of the left BA in a healthy brain, the right BA did not connect directly with the left WA. Instead it connected indirectly through the pre-SMA and the MFG (Li et al., 2019). Cho et al. reported reorganization of crossed cerebro-cerebellar activations (atypical cerebellar dominance) in right-handed patients harboring left frontal gliomas (Cho et al., 2018). The reorganization consisted of left-sided activity in the cerebellum (VI lobule) instead of the expected crossed activation, confirming the early report from Thiel et al. (2001). Similarly, Zhang et al. (2018) showed reorganization of cerebro-cerebellar language-related circuits in response to tumoral invasion for both LGG and HGG. Additionally, LGG patients exhibited gray matter volume changes in regions with increased brain activity, as per structural-functional coupling. The same was not true for high-grade tumors, which showed functional modifications only (Zhang et al., 2018). As most cited studies focused on LGG, few cases of inter-hemispheric reorganization in HGG have been described (Holodny et al., 2002; Petrovich et al., 2004; Gȩbska-Kośla et al., 2017). This may indicate that compensatory processes are a prerogative of gliomas as infiltrating lesions, irrespective of their grade. However, language reorganization in fast growing disruptive tumors has been poorly characterized and remains a matter of debate.

Despite high clinical relevance, very few studies have investigated language plasticity dependence on tumor location. Wang et al. (2013) reported that left frontal lesions may reduce functional activations of both BA and WA, while tumors in the left temporal lobe may affect WA alone. Furthermore, contralateral activations seem more consistent in tumors affecting the left frontal lobe. This finding suggested that BA plasticity is dependent on tumor location, while WA activation is dependent on individual patient characteristics (Buklina et al., 2015). On the other hand, different studies have reported the translocation of both BA and WA to the right side in cases of left-hemispheric glioma invading the frontal or temporal lobe (Kośla et al., 2012; Gȩbska-Kośla et al., 2017). Similarly, Partovi et al. demonstrated rightward reorganization of both BA and WA in response to tumors invading the respective dominant lobes (Partovi et al., 2012). In this case, the reorganization of BA was visible on phonemic fluency tasks and not on sentence completion, while the opposite was true for WA. This confirms the complementary role of the two covert speech tasks in language fMRI (Zacà et al., 2013; Black et al., 2017; Unadkat et al., 2019) and may suggest that discordant results about language reorganization depend partly on the employed task.

The effectiveness of right-sided activation for language—the ability to provide a reliable language function—is still debated. Few reports confirmed better clinical performance in patients with functional modifications consistent with reorganization (Kong et al., 2016; Shaw et al., 2016; Gȩbska-Kośla et al., 2017). Indeed, right-sided language areas proved to be functional by responding to virtual-lesions induced by transcranial magnetic stimulation (TMS) (Thiel et al., 2005; Krieg et al., 2013). In healthy volunteers, virtual-lesions predominantly disturbed language in the left hemisphere (ventral premotor cortex and the opercular part of the IFG), while language errors were elicited in both hemispheres for patients harboring left-hemispheric tumors. This suggests compensatory intra- and inter-hemispheric reorganization (Rösler et al., 2014). These studies reflect the evidence of patient recovery after resecting well-known eloquent areas (Russell et al., 2003; Lubrano et al., 2010; Sarubbo et al., 2012). On the other hand, contralesional activations did not always ensure optimal performance (Meyer et al., 2003a; Krieg et al., 2013; Cargnelutti et al., 2020).

Notably, functional plasticity may also be induced by surgery. Perilesional activation plays a pivotal role in language recovery after transient post-surgical language deficits (Kristo et al., 2015). For example, reorganization to nearby areas after surgery was demonstrated for the left lingual gyrus taking over the function of left parahippocampal gyrus in a picture-naming task (Deverdun et al., 2020). Similar to presurgical reorganization, resection of BA may lead to the recruitment of adjacent regions, in particular the ventral premotor cortex, pars orbitalis of the IFG, dorso-lateral prefrontal cortex, and insula, with better post-surgical language outcomes (Duffau, 2012). Insular tumors may activate the frontal and temporal operculum and the left putamen (Desmurget et al., 2007; Duffau, 2014). Resection of WA is associated with the recruitment of adjacent cortex and the progressive involvement of remote regions within the left-dominant hemisphere, including SMG, pars triangularis of the IFG or other left frontal-lateral regions, or contralateral homologues (Sarubbo et al., 2012). Similarly, resection of SMA may activate the contralateral SMA and premotor cortex (Krainik et al., 2004; Chivukula et al., 2018). Contralateral activations seem to support cognitive improvement in post-surgical reorganization as well, such as after glioma resection (Miotto et al., 2014).

### Clinical Relevance

Advances in treatment have increased life expectancy in patients with brain tumors. Consequently, long term preservation of quality of life has become a crucial focus of neuro-oncology. Consideration of language plasticity should therefore directly affect clinical care and therapeutic interventions of patients with brain tumors, including surgical approaches and timing. For example, multi-step surgical removal of LGG has been proposed to allow the development of plasticity-driven compensation of clinical deficits (Robles et al., 2008; Picart et al., 2019). The ability to accurately describe a specific hierarchy of active clusters on fMRI maps, characterized by a dominant cluster whose integrity is necessary for the stability of the network (del Ferraro et al., 2018; Li et al., 2020), may significantly implement preoperative planning of brain tumors. Furthermore, a better understanding of brain plasticity may allow enhancement of plastic phenomena to support surgical radicality. Preoperative cortical electrical stimulation and behavioral training in patients with low- and high-grade gliomas affecting eloquent areas may accelerate plastic changes (Rivera-Rivera et al., 2017), maximizing tumor resection and improving survival without post-surgical deficits.

Current knowledge suggests that both functional and structural modifications contribute to language plasticity in brain tumors. Integrating this growing knowledge into preoperative planning is a major field of research linking neuroscience to clinical neuroradiology.

## Language Plasticity Induced by Brain Ischemia

The study of structural and functional neuroplasticity in stroke is of particular interest, as the abrupt onset of the event gives investigators the opportunity to observe and characterize changes in the language network at different time points in the acute, subacute, and chronic stages. Moreover, studying brain plasticity after stroke may provide valuable information that predicts patient outcomes and may help to manage therapy. It is important to note that most plasticity studies regarding stroke tend to include both acute (first week after stroke) and subacute (from the second week onward) phases in a general “early phase,” as they share intense changes. We attempted to separate these two time points in this review for practical purposes.

### Acute Phase

**FIGURE 6:**
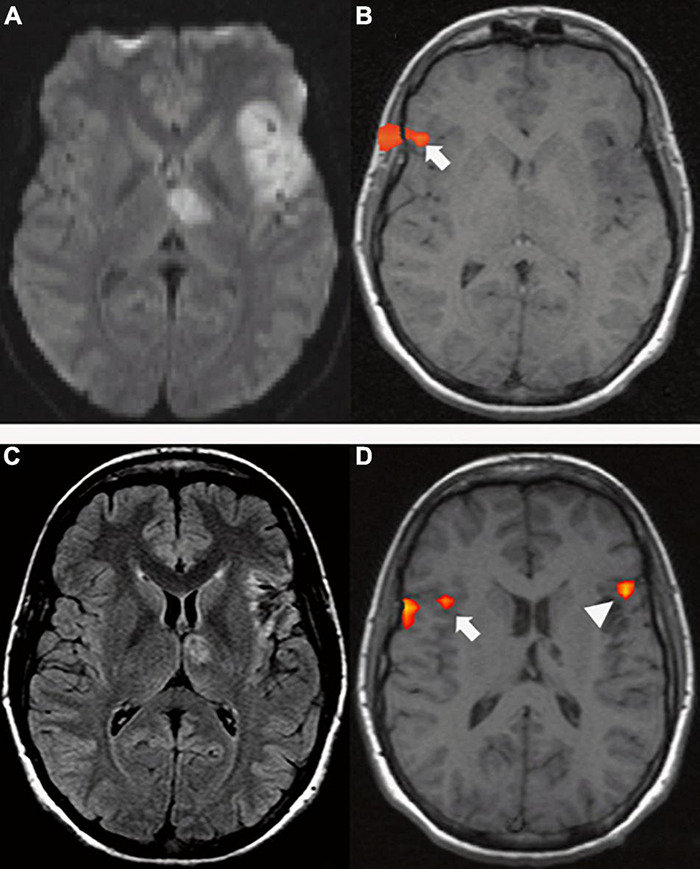
A 29-year-old right-handed woman with sudden onset of fluent aphasia. (Top row) MRI acquired in acute setting; **(A)** Axial DWI shows a left frontal and thalamic acute ischemia (due to patent foramen ovale); **(B)** tb-fMRI [verb generation task; *p* < 0.05; without restriction of cluster size (k = 0)] shows BOLD activation in the contralateral homotopic area (white arrow). The absence of significant activations in the left hemisphere could be caused by changes in the local blood flow secondary to ischemia. Functional activation at the level of the skull shell is related to mild motion artifact. Bottom row: MRI acquired four months after the event; **(C)** axial FLAIR shows post-ischemic changes in the left frontal and thalamic areas; **(D)** tb-fMRI [verb generation task; *p* < 0.05; without restriction of cluster size (k = 0)] shows BOLD activation in the left frontal area (white arrow head), as well as in the contralateral right frontal area (white arrow).

In the acute phase, aphasia is the result of both hypoperfusion and brain tissue damage (Hillis et al., 2001; Fridriksson et al., 2010a; Shahid et al., 2017). At this time, a bilateral reduced activation of the language network has been described and interpreted as global network disruption due to diaschisis (Saur et al., 2006). Diaschisis is a well-known phenomenon in which focal functional abnormalities caused by damage to critical nodes of the network and/or their connections are described in an anatomically intact region distant to the lesion (Carrera and Tononi, 2014; Hartwigsen and Saur, 2019). Indeed, disconnection has been found to be a mechanism of functional deficit in acute stroke patients (Corbetta et al., 2015). Frontal and temporo-parietal lesion sites have shown wide functional connectivity (FC) with remote brain areas on rs-fMRI (Hartwigsen and Saur, 2019), which explains how functional disconnection of distant areas can cause language deficits (Carter et al., 2012; Carrera and Tononi, 2014). Similar rs-fMRI modifications have been documented in posterior cerebral artery (PCA) stroke, as well (Sebastian et al., 2016). Concordantly, on structural imaging, acute language deficits have been found to be caused by disconnections due to white matter tract damage (Kümmerer et al., 2013). In particular, extensive connection damage is often caused by lesions of the temporal cortex, which has been demonstrated to be a highly connected hub (van den Heuvel and Sporns, 2011; Fornito et al., 2015). Diaschisis has been consistently demonstrated in the acute phase in patients with left temporo-parietal strokes as a lack of ipsilesional frontal activation. On the other hand, patients with left frontal stroke tend to show preserved left temporal and right frontal activation along with right homotopic language areas (Figure 6; Stockert et al., 2020).

One of the most clinically interesting challenges of neuroimaging plasticity studies is the effort to predict patient outcomes from functional and structural imaging parameters acquired in the early phases of strokes. For example, Saur et al. (2010) aimed at predicting the degree of language improvement over six months after left-hemispheric stroke by applying a multivariate machine learning technique to fMRI data. In the acute phase, diaschisis affected the activation of most language-related areas and reduced prediction performance, with the exception of primary auditory areas that predicted outcome at six months. This is likely due to intrinsic resistance to diaschisis (Saur et al., 2010). Another interesting application of fMRI is the evaluation of treatment-induced plasticity to better understand the causes of therapy success or failure. This knowledge would increase the strategic efficacy of therapy. Currently, only the study by Mattioli et al. (2014) has explored this field in the acute stage after stroke. The study found that rehabilitation in the early phases is correlated with increased recruitment of left IFG on tb-fMRI, which lasted over time and improved object naming (Mattioli et al., 2014). A single-case study reported the beneficial role of early therapeutic intervention by monitoring event-related potentials (ERPs) at electroencephalography (EEG), which have been proven to be useful in monitoring language abilities in aphasia patients (Aerts et al., 2015). Other recent clinical studies showed no deficit improvement after early aphasia treatment (Coleman et al., 2017; Nouwens et al., 2017).

During the acute phase, fMRI studies are hindered by several limitations. First, therapy-induced plasticity is sometimes difficult to separate from normal recovery due to the intense changes that occur immediately after stroke. Second, but equally importantly, tb-fMRI is often highly demanding for people with acute aphasia, especially with severe impairment, and results in difficult acquisition and data elaboration. rs-fMRI and structural imaging could be useful in this analysis, as they do not require strict collaboration from the patient. However, such studies have yet to be published. Further research is needed to verify how early functional and structural plasticity measures are associated with rehabilitation improvement and patient outcomes. In particular, fMRI activation differences based on lesion location should be explored in light of new discoveries regarding diaschisis soon after stroke (Stockert et al., 2020).

### Subacute Phase

With spontaneous recovery in the subacute phase, upregulation of homologous right-hemispheric language areas and reactivation of perilesional cortex and left cerebellar areas is observed (Saur et al., 2006; Nenert et al., 2018; Stockert et al., 2020). Diaschisis eventually resolves, although the timing varies among cases (Stockert et al., 2020). In healthy subjects, the right cortex interprets non-linguistic characteristics of language, such as change of prosody and letter size (Baumgaertner et al., 2013). On a structural level, connections between right language areas parallel those of left language areas (Kaplan et al., 2010). This could explain the early recruitment of right homotopic language regions, as they seem to be predisposed to language function.

Furthermore, extensive recruitment of domain-general control systems is observed regardless of the lesion site. This is especially true of the pre-supplementary motor area and dorsal anterior cingulate cortex (pre-SMA/dACC) (Brownsett et al., 2014; Geranmayeh et al., 2014). When considering rs-fMRI, in subacute phase, FC is found to be decreased in networks (both intra- and inter-hemispheric) that are usually integrated. Conversely, FC is found to be increased in networks that are usually segregated (Siegel et al., 2016, 2018). In the language domain, both FC and lesion topography predicted behavioral variance. Moreover, language impairment was predicted by both inter-hemispheric connections between networks and left intra-hemispheric FC (Siegel et al., 2016). These results are in-line with previous studies that found a relationship between altered inter-hemispheric FC and aphasia severity (Zhu et al., 2014). These support the conception of aphasia as a network disorder because language functions rely on highly specialized areas, bilateral networks, and connections (Fedorenko and Thompson-Schill, 2014). Accordingly, in subacute PCA stroke, as naming performance recovers, intra- and inter-hemispheric ipsilesional and contralesional language cortex FC increases (Sebastian et al., 2016). This same trend is confirmed in an EEG study that found decreased inter-hemispheric phase synchrony indexes as a common response in subacute stroke (Kawano et al., 2021).

During the subacute phase, language fMRI was shown to be a useful outcome predictor. Saur et al. demonstrated that adding fMRI language activation data (acquired in the second week after stroke) to a patient’s initial impairment and age predicted language deficit more accurately after six months than did initial deficit and age alone (Saur et al., 2010). Furthermore, activity level of pre-SMA/dACC approximately two weeks after stroke has been shown to be positively correlated with aphasia recovery at four months (Geranmayeh et al., 2017). The recruitment of these areas may help people with aphasia to overcome disconnections with other brain regions. It has been previously correlated with better out-of-scanner picture description performance, as well (Brownsett et al., 2014). Recruitment of different brain areas, including the non-dominant hemisphere, is also found to be a predictor of aphasia recovery on EEG (Nicolo et al., 2015; Kawano et al., 2021).

As opposed to functional imaging studies, structural imaging studies focus on individual white and gray matter structures as a predictor of patient outcomes rather than on the entire network. Damage to the dominant temporal cortex and underlying white matter has been correlated with poorer aphasia outcomes (Dronkers et al., 2004), especially in areas corresponding with the superior and long segment of AF, SLFII, and ILF (Ramsey et al., 2017). Accordingly, Kim and Jang found that patients whose AF could not be reconstructed on DTI tractography at the acute stage had worse aphasia outcomes (Kim and Jang, 2013). This is in-line with another study that correlated acute damage to left STG and/or SLF/AF with worse naming skill recovery (Hillis et al., 2018). Moreover, recovery prediction at six months was found to improve by adding the volume of the long segment of the left AF (but not other tracts—e.g., FAT or IFOF) to a regression model based on age, sex, and lesion size (Forkel et al., 2014; Forkel and Catani, 2018). The inclusion of the long segment of the contralesional AF improved the prediction even more (Forkel et al., 2014). These studies underline the importance of the contralesional hemisphere in post-stroke plasticity even in early phases. Researchers discovered the compensatory mechanism of right language homotopic areas on structural MRI to be in agreement with functional studies (Hillis and Heidler, 2002; Crinion and Price, 2005; Saur et al., 2006; Price et al., 2017; Stockert et al., 2020). However, another study did not find this compensation to be important in recovery, but found that left fronto-parietal regions drive the process (Nenert et al., 2018).

Future directions could combine both structural and functional imaging to better comprehend the interplay between areas of activation, brain networks (both at rest and during tasks), and white matter tracts in aphasia recovery. Considering the studies that support domain-general networks as predictive of language recovery (Fedorenko and Thompson-Schill, 2014; Geranmayeh et al., 2014, 2017), these areas could be targeted for future therapies, especially in temporo-parietal stroke, which has been correlated with a stronger global network disturbance (Stockert et al., 2020).

### Chronic Phase

Both spontaneous and treatment-induced plasticity during the chronic phase of stroke have been investigated due to the larger number of available patients who are usually more compliant with studies than are patients in the early period after stroke. In this stage, all intensive changes that occurred in the early phases (from diaschisis to resolution of network disruption and structural disconnection) recede and yield to intrinsic network reorganization whereby different components replace the functions of the damaged area (Jarso et al., 2013). Functional recovery is dependent on different factors and processes important for network reorganization, including lesion volume and location, vascular physiology, spared cortical areas, and white matter integrity (Kiran and Thompson, 2019).

One of the cornerstone models of aphasia recovery after stroke is the hierarchical model proposed by Heiss and Thiel (2006). These authors stated that the best aphasia outcome is associated with small left side lesions, implying fewer disconnections among language-dominant left hemisphere regions. Satisfactory recovery can be obtained after perisylvian language cortical damage by recruiting extrasylvian ipsilesional brain regions. Finally, poor behavioral recovery is associated with wide left hemisphere lesions, where only extensive right hemisphere recruitment can take over language production (Heiss and Thiel, 2006). Several tb-fMRI studies consistent with this model demonstrated that patients with the best language performance after stroke recruited the left hemisphere cortex and perilesional areas during language tasks (Fridriksson, 2010; Fridriksson et al., 2010a,2012b; van Oers et al., 2010; Szaflarski et al., 2013; Robson et al., 2014; Griffis et al., 2017). The contribution of the right hemisphere in language recovery is debated and is thought to be associated with poor language production in the chronic phase as a result of maladaptive plasticity (Heiss and Thiel, 2006; Saur et al., 2006). This concept is supported by functional imaging studies that correlated right hemisphere activation with poor language outcomes (Blank et al., 2003; Naeser et al., 2004; Richter et al., 2008; Postman-Caucheteux et al., 2010) and by non-invasive brain stimulation (NIBS) studies that showed improved language performance after inhibition of the right hemisphere (Naeser et al., 2005; Tsai et al., 2014; Rubi-Fessen et al., 2015). Conversely, other studies have reported prolonged right (or bilateral) hemisphere activation to be associated with good language skills (Mattioli et al., 2014; Mohr et al., 2014). Furthermore, activation of bilateral domain-general networks has been found to play an important role in the recovery of stroke patients (Brownsett et al., 2014; Fedorenko and Thompson-Schill, 2014; Geranmayeh et al., 2014, 2017). It has been suggested that these areas, much like the right language homotopic regions, are recruited as compensation in the chronic phase. Once again, right activation is particularly visible in left IFG stroke, which is associated with bilateral IFG activation (Turkeltaub et al., 2011). It is interesting to notice that different electrophysiology studies agree with the findings of positive right hemisphere contribution to aphasia recovery (Mielke and Szelies, 2003; Pulvermüller et al., 2005; Lucchese et al., 2017; Piai et al., 2017). In particular, Piai et al. suggest a role in language compensation of posterior transcallosal white-matter connections through the splenium (Piai et al., 2017). The recruitment of right language homotopic areas and domain-general networks and their active role in language recovery may be explained as language reorganization, which has been demonstrated to occur in pre-existing networks (regardless of the lesion site), as well as outside traditional language networks (Turkeltaub et al., 2012; Nenert et al., 2018; Stockert et al., 2020). It is likely that speaking of the “right hemisphere” as a whole is reductive, as it has been argued that some areas facilitate language production while others may interfere with recovery (Turkeltaub et al., 2012).

Nonetheless, it is important to remark on the centrality of the left hemisphere, often the center of reorganization of linguistic functions (Nolfe et al., 2006; Spironelli et al., 2008). Structural imaging investigations have correlated clinical symptoms with necrosis of the cortex (Fridriksson et al., 2016; Pillay et al., 2017) and the underlining disruption of structural connection (white matter integrity). These are shown to be important negative factors in language recovery (Bonilha et al., 2014, 2017; Gleichgerrcht et al., 2015; Yourganov et al., 2016). For instance, one study found reduced FA in the left different white matter tracts after stroke. Particularly, the AF, IFOF, and ILF correlated with sentence and word comprehension to varying degrees (Ivanova et al., 2016). Furthermore, lesion load in the AF predicted speech efficiency and naming ability (Marchina et al., 2011). These results are concordant with rs-fMRI analyses that observed reduced ipsilesional intra- and inter-hemispheric connectivity in chronic aphasia patients (Sandberg, 2017). However, another DTI study focused on the importance of the right hemisphere, correlating high FA values of these areas with improved language recovery (Pani et al., 2016). Other studies have demonstrated an increase in right-hemispheric local gray matter volume to be correlated with better language production (Xing et al., 2016; Hope et al., 2017).

A recent study using fMRI and dynamic causal modeling in stroke patients found that damage to specific left hemisphere structures led to increased right or left intra-hemispheric connectivity depending on the location. The authors also observed increased inter-hemispheric connections with a semantic task, possibly due to broader network recruitment compensating for structural and/or functional disconnection. Therefore, the study concluded that the chronic recovery process cannot be attributed only to one hemisphere vs. the other (Meier et al., 2019). Accordingly, interactions between different networks are a better predictor of language production than activity within a single network, highlighting the diffuse distribution of this complex process (Geranmayeh et al., 2016).

**FIGURE 7:**
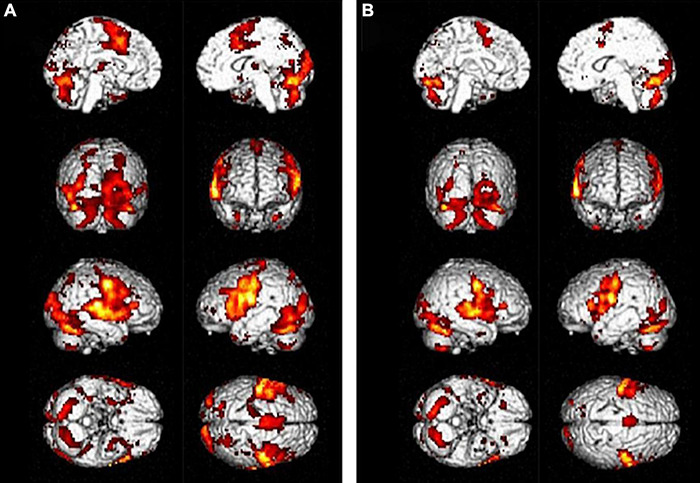
A 47-year-old woman with chronic (>six months) left ischemia and naming aphasia. **(A)** tb-fMRI [naming task; *p* < 0.05; FEW corrected (T = 4.70); without restriction of cluster size (k = 0)] shows high BOLD bilateral activation, especially in the right frontal area; **(B)** after anomia treatment, naming tb-fMRI [*p* < 0.05; FEW corrected (T = 4.70); without restriction of cluster size (k = 0)] shows reduced BOLD activation with particular reference to the right hemisphere BOLD signal. These results were interpreted as neural priming with better neural efficient associated with less effort. Courtesy of Nardo et al. (2017) Brain 2017; funded by Wellcome Trust Senior Research Fellowship in Clinical Science (106161/Z/14/Z); MRC Clinical Scientist Fellowship (G0701888).

Treatment-induced plasticity in the chronic phase has demonstrated the contribution of both the left and right hemispheres to language recovery. Many studies have observed therapy-related improvement to be associated with decreased right-hemispheric activation (Richter et al., 2008; Abel et al., 2015; Nardo et al., 2017). Interestingly, in one study, pre-treatment right-hemispheric activation was positively correlated with subsequent therapy success (Richter et al., 2008). These observations could underlie a facilitatory role of the right hemisphere due to more efficient task processing, as for neural priming, meaning that increased BOLD signal does not correspond to improved efficiency (Figure 7; Nardo et al., 2017). Comparably, Abel et al. (2015) demonstrated that therapeutic success correlated only with activation decreases in the right hemisphere. Contribution of the right hemisphere after therapy is also supported by a DTI study that found a correlation between right AF volume increase and improvement after melodic intonation therapy (Schlaug et al., 2009). Activation decrease with increased task processing was also found in the left hemisphere regions (Fridriksson et al., 2012a).

In contrast, many other investigations correlated increased activation in the right hemisphere with improvement after therapy. For instance, when using a picture-naming treatment specifically designed to elicit right frontal cortex activation, a left-to-right lateralization associated with treatment success was observed (Crosson et al., 2005); this was maintained in follow-up scans and showed behavioral generalization to untrained objects as well (Crosson et al., 2009; Benjamin et al., 2014). Activation was found in the left peri-lesional cortex (Fridriksson et al., 2010b; Mattioli et al., 2014; van Hees et al., 2014a; Zhu et al., 2014), in bilateral regions (Pulvermüller et al., 2005; Fridriksson et al., 2006; Brownsett et al., 2014), and during rs-fMRI (van Hees et al., 2014b). Finally, Haldin et al. (2018) used a sensori-motor rehabilitation method in one patient and obtained language improvement associated with increased activity in brain areas outside the language network (i.e., somatosensory areas). Once again, this highlights the complexity of the language process. Finally, the contribution of both hemispheres in language recovery is highlighted by electrophysiological studies (both MEG and EEG) (Pulvermüller et al., 2005; Mohr et al., 2016; Lucchese et al., 2017).

These contrasting results are not necessarily contradictory, as brain region recruitment may vary according to lesion location and size (Hamilton et al., 2011). Supporting this contention, the results of an independent component analysis by Abel et al. (2015) found decreased activations to be dependent on lesion site. In addition, aphasia type, treatment strategy, intensity, and even difficulty are important factors that influence task-related activity (Hartwigsen and Saur, 2019). For example, using MEG, Mohr et al. (2016) found contributions from both hemispheres in therapy-induced aphasia recovery, but with stronger left lateralization. They justified this result with the administration of a passive listening paradigm to their subjects, thus requiring too few processing demands to elicit right hemisphere circuits (Mohr et al., 2016). Furthermore, it is important to emphasize that brain area recruitment may differ among individuals, as white matter tracts and the behavioral effect of therapies vary (Kiran and Thompson, 2019). Finally, increased activity may imply increase task-demands and disinhibition, as well as eventual successful or maladaptive reorganization (Naeser et al., 2005; Abel et al., 2015).

Future studies should use a multimodal approach with both structural and functional imaging to shed light on the correlation between brain area activation and white matter tract integrity in treatment-induced plasticity, taking account of the variability induced by lesion location and size. In addition, both rs-fMRI and tb-fMRI methods are advocated to better understand how networks change in response to rehabilitation.

### Clinical Relevance

As new research increases our knowledge of this complex and delicate process involving multiple brain areas and networks, clinical applications have become more plausible. From outcome prediction based on tract damage and network disturbance, to the targeting of specific areas in different phases to help language recovery, neuroradiologists should be aware of these processes and tailor approaches to their patients.

## Language Plasticity Induced by Epileptic Foci

Chronic epilepsy is associated with prolonged, low-intensity injury to the brain, which may induce reorganization of language. It is now clear that epilepsy is a network disorder, where epileptic activity induces alteration of neural connectivity (de Palma et al., 2020). EEG data have shown that neural networks in epileptic patients reorganize both within the epileptogenic area and into normal functional neural circuits beyond (Schevon et al., 2007). In this complex scenario, the definition of language dominance is of pivotal importance to the assessment of risk-benefit balance during surgical planning of an epileptic patient. In the last decades, different non-invasive techniques beyond fMRI such as positron emission tomography (PET) and MEG have also been exploited to study the definition of hemispheric dominance and evaluate language networks in both healthy and affected brains (Pirmoradi et al., 2010; D’Arcy et al., 2013; Balter et al., 2019).

**FIGURE 8:**
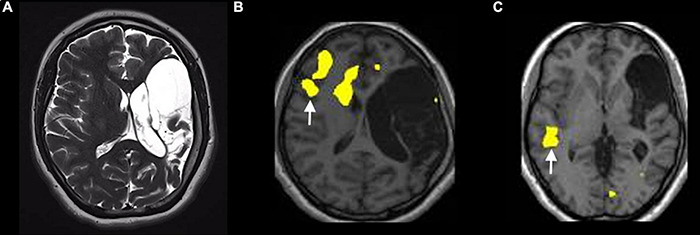
Example of atypical lateralization in a patient with drug-resistant epilepsy from perinatal ischemic lesion, displayed on a verb generation task [*p* < 0.05; FEW corrected; without restriction of cluster size (k = 0)]. **(A)** T2-weighted images on the axial planes show a large area of cystic encephalomalacia in the outcome of left frontal-insular-parietal perinatal infarction. The fMRI exam performed with a verb generation task **(B)** documents the activation of right frontal areas, including the inferior frontal gyrus [arrow in panel **(B)**]. The story listening task **(C)** documents the activation of right a temporal area [arrow in panel **(C)**], representing Wernicke’s area homologous.

Still, it is not clear if atypical language in epileptic patients is the result of reorganization, compensation, or abnormal development of language functions. Epileptic activity is often triggered by focal lesions such as brain tumors or stroke (Figure 8). In these cases, it is difficult to separate language plasticity induced by epileptic activity from the effect of an underlying lesion. Some studies suggest that epileptic activity, especially in the left hemisphere, may cause language impairment independent from the presence of an underlying lesion (Croft et al., 2014). In an fMRI study on children with left focal epilepsy and an unremarkable MRI, the patient group demonstrated lower verbal abilities than healthy controls, which is associated with decreased activation of ventral language regions across childhood (Croft et al., 2014). Such network perturbations may be associated with language plasticity in certain cases.

As in previously discussed conditions, language reorganization in epileptic patients can follow different models: (a) inter-hemispheric reorganization, in which the spectrum of lateralization is shifted to the contralateral hemisphere and activation is seen in right-sided homologues of dominant language areas (Rosenberger et al., 2009); (b) inter-hemispheric dissociation of linguistic sub-functions (cross dominance), in which the areas for verbal production and verbal comprehension are located in different hemispheres (Balter et al., 2019); and (c) intra-hemispheric reorganization, in which the language functions reorganize close to typical language zones in the frontal and temporal lobes (Mbwana et al., 2009). However, these models are thought to oversimplify reality (Berl et al., 2015). There are several predictors of atypical language in epileptic patients. Clinical variables have been shown to contribute to language reorganization in chronic seizure disorders, yet they failed to reliably predict altered language networks on an individual basis (Hamberger and Cole, 2011). Age at onset is considered a possible predictor of language representation, although not univocally. Some studies have suggested that seizure onset before six years of age may predict language reorganization, yet other studies have not reproduced this result (Janszky et al., 2003; Liégeois et al., 2004; Berl et al., 2015).

Atypical language representation has been observed in patients suffering from temporal lobe epilepsy (TLE), especially when the epileptic focus originates from the language-dominant hemisphere (Adcock et al., 2003; Brázdil et al., 2005; Chang et al., 2017; Balter et al., 2019). Furthermore, patients with left mesial temporal sclerosis may display language reorganization involving both the temporal and frontal areas. This evidence confirms the participation of the hippocampus in the language network and suggests that early insult to the hippocampal-verbal working system may lead to reorganization of language regions (Pataraia et al., 2004; Weber et al., 2006; Berl et al., 2015). Functional plasticity in epileptic patients can also be evaluated with network-based approaches (Vlooswijk et al., 2010; Karunanayaka et al., 2011; Eddin et al., 2014). Graph-theoretical analysis has been applied to investigate language function in epileptic children undergoing an auditory word definition decision task (Eddin et al., 2014). Compared to controls, patients exhibited a less efficient language network, as demonstrated by a larger extent and lesser compartmentalization of the activated network. Interestingly, the reduction of language network function was not related to hemispheric lateralization, suggesting that the deficit is independent from the presence of atypical language organization.

In addition to functional reorganization, epileptic patients may show structural changes. A multimodal approach using neuropsychology, fMRI, and DTI has shown left-to-right shift fMRI activation associated with reduced asymmetry in DTI parameters of the AF in patients with left TLE when compared to controls. These results indicate a trend toward inter-hemispheric reorganization of both cortical regions and subcortical white matter tracts in the language patterns of left TLE affected patients (Chang et al., 2017). Using a machine learning approach, Paldino et al. (2014) demonstrated significant differences between FA and mean diffusivity (MD) values in the language-associated white matter tracts of epileptic children with language impairment compared to those with preserved language function. Specifically, the authors detected differences in DTI parameters of the left AF, IFOF, and UF, strengthening the idea that multiple fibers may contribute to the overall connectivity of the language network. Furthermore, the same authors reported a significant correlation between left AF fiber-tracking and the presence of language deficits in epileptic children with malformations of cortical development (Paldino et al., 2016). In the same patients, the lack of identification of the right AF was associated with a complete absence of oral language, suggesting an important role of inter-hemispheric white matter plasticity for language function preservation. On the other hand, Labudda et al. (2012) demonstrated cortical thickening of right-sided language area homologues in patients with epileptic foci in the left hemisphere, reaffirming that focal lesions may trigger both cortical and subcortical structural plasticity.

### Clinical Relevance

Preoperative definition of language lateralization is of fundamental importance to postoperative cognitive outcomes for epileptic patients. After left anterior temporal lobe resection for TLE, patients with left dominant hemisphere often exhibit reduced verbal learning and memory deficits. Indeed, several studies have demonstrated that patients with right or bilateral hemisphere language representation show better verbal memory performance after temporal lobe resection than patients with left dominant hemisphere (Gleissner et al., 2003; Fatoorechi et al., 2020). Therefore, it is intuitive to consider atypical language organization as a protective factor for postoperative language preservation.

## Comparing Plasticity Induced by Focal Lesions

**FIGURE 9:**
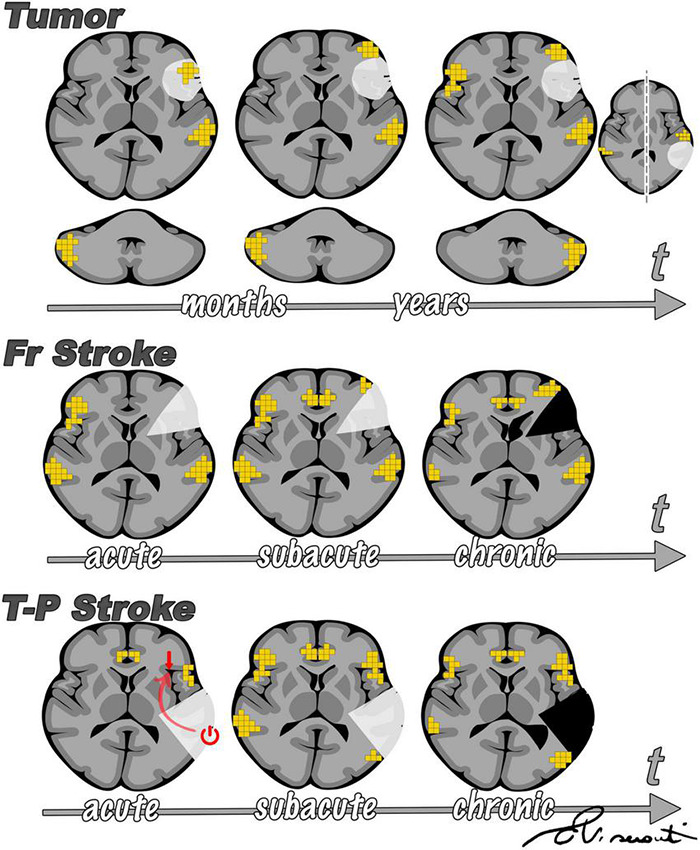
Summary of main differences emerging from the comparison of sudden onset (such as stroke) vs. slow onset focal lesions (such as tumors). The figure is intended as a summary of the results of several research studies discussed throughout this review, in line with our observations. In brain tumors **(upper panel)**, fMRI activity is first observed inside the lesion, then in the perilesional tissue, and only later in the contralesional hemisphere. Such findings were proposed by Desmurget et al. (2007) and supported by other studies. Cerebellar activation has been reported to reorganize from a normal contralateral representation to homolateral, as supported by findings from our group (Cho et al., 2018). According to prior studies (Saur et al., 2006), after stroke **(central and lower panel)** there is initial left-to-right shift of fMRI activation and a progressive return to the left perilesional area, with support of domain-general regions. Diaschisis has more frequently been described arising from temporal-parietal stroke than from frontal lesions (Stockert et al., 2020), influencing language representation in the acute phase **(lower panel)**.

Comparing the effect of different focal lesions offers interesting insights into the language network and its reorganization in response to an insult. Here we present a summary of language plasticity related to different focal lesions as inferred from previously published studies, including our own research experience, which is in line with our clinical observations (Figure 9).

Stroke and brain tumors exemplify two opposite poles for the onset of a focal lesion. The primary difference between tumor- and stroke-induced plasticity is the longitudinal development of language reorganization. In low-grade tumors, fMRI activity is first observed inside the lesion, next in the perilesional tissue, and only later in the contralesional hemisphere. However, after stroke there is an immediate left-to-right shift of fMRI activation and a progressive return to the left perilesional area with support of domain-general regions in later phases (Saur et al., 2006; Geranmayeh et al., 2014; Stockert et al., 2020). One explanation may lie in the abrupt onset of stroke. After a sudden loss of function, the brain may attempt to rely on areas that are structurally (Kaplan et al., 2010) and functionally (Baumgaertner et al., 2013) similar for compensation. Conversely, when a lesion is slowly invading functional areas, as in tumors, the brain may have the time to reorganize networks to guarantee the best functional performance. These dynamics are well-described by neuro-computational models (Keidel et al., 2010). In addition, neuroplasticity processes require an increase of CBF with intense enzyme cascade that could be blocked by the severe hypoperfusion that occurs in the penumbra right after stroke (Hillis et al., 2001). Unfortunately, hypoperfusion hinders our ability to explore brain functions during this phase because the detection of brain activity on fMRI relies on the blood oxygenation level. Another interesting point is that the brain reorganizes differently depending on lesion location. Contralesional (right) activation is more likely to occur in both stroke and tumor in the left frontal lobe than in tumor of the temporal lobe. Diaschisis has more frequently been described in temporal-parietal stroke than in frontal lesions (Stockert et al., 2020). Accordingly, adaptive contralateral plasticity involves BA more often than WA in brain tumors (Wang et al., 2013; Buklina et al., 2015; Gȩbska-Kośla et al., 2017). This may imply a functional dependency of the frontal on the temporal cortex, as the latter is believed to serve as a hub to integrate auditory stimuli with other higher-order processes (Lau et al., 2008). Studies conducted on epileptic patients may increase our understanding of age-related aspects of language plasticity. fMRI studies have demonstrated that language reorganization is more frequent in children affected by epilepsy than adults, thus corroborating the idea that the developing brain is more prone to reorganization (Liégeois et al., 2004).

## Conclusion

Language plasticity remains a puzzling question in modern neuroscience, with intriguing clinical applications. Lesion dynamics influence the manifestation of plasticity, as depicted by neuroimaging studies. In particular, the timing of lesion occurrence can affect reorganization and result in different radiological features. Language plasticity must be evaluated in the context of modern network theories of cognitive function that involve both white matter structural changes and functional modifications. Currently, we employ comparable tools to investigate mind processes as to perform routine clinical examinations. fMRI stands between experimental cognitive neuroscience and routine patient care, such as preoperative planning of brain lesions. Graph theory and connectomics will provide important insights into network dynamics and plasticity triggered by brain lesions. Eventually, this knowledge may lead to personalized therapeutic treatments based upon individual patients’ susceptibility to plastic compensation for clinical deficits.

## Author Contributions

LP and AD: conceptualization and project administration. LP, AD, MR-E, EV, and KP: data curation. LP, AD, and MR-E: methodology and writing—original draft. AN, AH, KP, AB, and AR: resources and supervision. All authors writing—review and editing.

## Conflict of Interest

AH is the Owner/President of fMRI Consultants, LLC, a purely educational entity. The remaining authors declare that the research was conducted in the absence of any commercial or financial relationships that could be construed as a potential conflict of interest.

## Publisher’s Note

All claims expressed in this article are solely those of the authors and do not necessarily represent those of their affiliated organizations, or those of the publisher, the editors and the reviewers. Any product that may be evaluated in this article, or claim that may be made by its manufacturer, is not guaranteed or endorsed by the publisher.

## Abbreviations

AF: arcuate fasciculus
AG: angulate gyrus
ASFNR: American Society of Functional Neuroradiology
BA: Broca’s area
BOLD: blood oxygenation level dependent
CBF: cerebral blood flow
COX-2: cyclooxygenase 2
CP: critical period
dACC: dorsal anterior cingulate cortex
DCS: direct cortical stimulation
DTI: diffusion tensor tractography
EA: Exner’s area
EEG: electroencephalography
ERP: event-related potential
FA: fractional anisotropy
FAT: frontal aslant tract
FC: functional connectivity
fMRI: functional magnetic resonance imaging
GABA: gamma-aminobutyric acid
HGG: high grade glioma
IDH: isocitrate dehydrogenase
IFG: inferior frontal gyrus
IFOF: inferior frontal-occipital fasciculus
ILF: inferior longitudinal fasciculus
ITG: inferior temporal gyrus
LGG: low grade glioma
LTD: long term depotentiation
LTP: long term potentiation
MEG: magnetoencephalography
MFG: middle frontal gyrus
MTG: middle temporal gyrus
NIBS: non-invasive brain stimulation
nNOS: neuronal NO synthase
NODDI: neurite orientation dispersion and density imaging
PCA: posterior cerebral artery
PET: positron emission tomography
PreMA: premotor area
PVZ: periventricular zone
Rs-fMRI: resting-state functional magnetic resonance imaging
SFG: superior frontal gyrus
SLF: superior longitudinal fasciculus
SMA: supplementary motor area
SMG: supramarginal gyrus
STG: superior temporal gyrus
Tb-fMRI: task-based functional magnetic resonance imaging
TLE: temporal lobe epilepsy
TMS: transcranial magnetic stimulation
UF: uncinate fasciculus
VBM: voxel-based morphometry
VWFA: visual word form area
WA: Wernicke’s area.
